# Polymorphic patterns of the merozoite surface protein-3β in Korean isolates of *Plasmodium vivax*

**DOI:** 10.1186/1475-2875-13-104

**Published:** 2014-03-17

**Authors:** Jung-Mi Kang, Hye-Lim Ju, Pyo Yun Cho, Sung-Ung Moon, Seong Kyu Ahn, Woon-Mok Sohn, Hyeong-Woo Lee, Tong-Soo Kim, Byoung-Kuk Na

**Affiliations:** 1 Department of Parasitology and Tropical Medicine, and Institute of Health Sciences, Gyeongsang National University School of Medicine, Jinju 660-751, Republic of Korea; 2 Department of Parasitology and Inha Research Institute for Medical Sciences, Inha University School of Medicine, Incheon 400-712, Republic of Korea; 3 Department of Internal Medicine, Seoul National University Bundang Hospital, Seongnam 463-707, Republic of Korea; 4 Department of Pathology, Immunology, and Laboratory Medicine, College of Medicine, University of Florida, J-566, 1275 Center Drive, Gainesville, FL 32610, USA

**Keywords:** *Plasmodium vivax*, Merozoite surface protein-3β, Genetic polymorphism, Natural selection, Korea

## Abstract

**Background:**

The merozoite surface protein-3β of *Plasmodium vivax* (PvMSP-3β) is one of the candidate antigens for blood stage malaria vaccine development. The polymorphisms in PvMSP-3β have been reported in certain *P. vivax* isolates. However, the diversity of PvMSP-3β throughout its global distribution has not been well understood. In this study, the genetic diversity and the effects of natural selection in PvMSP-3β among *P. vivax* Korean isolates were analysed.

**Methods:**

Blood samples were collected from 95 patients with vivax malaria in Korea. The region flanking full-length PvMSP-3β was amplified by polymerase chain reaction and cloned into a TA cloning vector. The PvMSP-3β sequence of each isolate was determined and the polymorphic characteristics and effects of natural selection were analysed using the DNASTAR, MEGA4, and DnaSP programs.

**Results:**

Five different subtypes of PvMSP-3β were identified based on single nucleotide polymorphisms (SNPs), insertions, and deletions. Although a high level of sequence diversity was observed in the PvMSP-3β gene, the coiled-coil tertiary structure of the PvMSP-3β protein was well conserved in all of the sequences. The PvMSP-3β of Korean isolates is under natural selection. DNA polymerase slippage and intragenic recombination likely contributed to PvMSP-3β diversity in Korean *P. vivax* isolates.

**Conclusions:**

The PvMSP-3β of Korean *P. vivax* isolates displayed polymorphisms, with SNPs, insertions and deletions scattered throughout of the gene. These results of parasite heterogeneity are relevant to the development of a PvMSP-3β based vaccine against *P. vivax* and the implementation of malaria control programmes in Korea.

## Background

*Plasmodium vivax* is the most widely distributed malaria parasite in worldwide, causing 70–80 million cases annually, and is a major public health problem contributing to the global burden of malaria morbidity in regions outside of Africa [1]. *Plasmodium vivax* is more difficult to control and eliminate than *Plasmodium falciparum* mainly because of its tendency to relapse. Moreover, vivax malaria has re-emerged in many temperate regions in recent years, including South Korea, where it had been largely eradicated during the global malaria control campaigns.

Understanding the population structures of the malaria parasites is an important factor in drug-resistance surveillance and estimating the performance of vaccines under development in a particular parasite population [2]. Although the population structures of *P. falciparum* isolates have been examined on a global scale, those of *P. vivax* remain largely less understood. With a global distribution that ranges from tropical to temperate regions, *P. vivax* isolates have exhibited variation in biological characteristics, such as relapse patterns and transmissibility, which can be used to distinguish geographical isolates and even subspecies [3]. Moreover, extensive genetic diversity has also been identified within a local *P. vivax* population. Therefore, understanding the nature of genetic polymorphisms of *P. vivax* populations in endemic areas might provide information regarding parasite heterogeneity that is relevant to malaria control efforts.

The *P. vivax* merozoite surface protein-3 (PvMSP-3) family consists of several related proteins, PvMSP-3α, PvMSP-3β and PvMSP-3γ. Although the level of shared amino acid sequence identity among the proteins is limited, all of them contain a central alanine-rich domain that forms a coiled-coil tertiary structure [4-6]. Limited sequence polymorphism has been observed in the MSP-3 of *P. falciparum* (PfMSP-3) [7,8], but the PvMSP-3α is likely to be highly polymorphic and is reported to be a reliable genetic marker for population analysis [9-13]. PvMSP-3β also showed polymorphic patterns among several geographically different *P. vivax* isolates [14,15], but its polymorphic pattern in worldwide isolates is poorly understood compared to other vaccine candidate antigens. As PvMSP-3β has been regarded as a potential vaccine target [16], a more thorough understanding of genetic diversity of the gene is required since genetic polymorphism in the candidate antigen can hamper the efficacy of a vaccine.

Following its re-emergence in Korea in 1993, vivax malaria has persisted, with varying numbers of indigenous cases annually in the country. Several recent studies have suggested that rapid genetic variation has occurred in the Korean *P. vivax* population in recent years [17-21], which supports settlement of local transmission of vivax malaria in Korea. In this study, the polymorphic nature of PvMSP-3β in Korean *P. vivax* isolates was analysed. The PvMSP-3β gene is radically polymorphic in Korean *P. vivax* population, with multiple gene sizes and single nucleotide polymorphisms (SNPs) which are scattered throughout the gene.

## Methods

### Blood samples

A total of 95 blood samples used in this study were collected from Korean patients infected with *P. vivax* in Korea between 2007 and 2012 (2007–2010, *n* = 15 for each year; 2011, *n* = 18; 2012, *n* = 17). All the patients inhabited in malaria endemic areas, Ilsan, Kimpo or Yonchon, and have not been abroad at least in recent 2 years when their blood samples were collected. The *P. vivax* infections were identified by microscopic examination of thin and thick blood smears, and confirmed by polymerase chain reaction (PCR) [10,20]. About 5 ml of blood was collected from each individual. The blood was separated into packed cells and plasma and then stored at −80°C until use. Informed consent was obtained from all of the patients before blood collection. The study protocol was approved by the Ethics Committee of the Inha University School of Medicine.

### Genomic DNA extraction and amplification of PvMSP-3β

Genomic DNA was extracted from 200 μl of whole blood sample using the QIAamp DNA Blood Kit (Qiagen, Hilden, Germany). The full-length region encoding PvMSP-3β was amplified using two rounds of PCR. The primers were designed based on PvMSP-3β sequences of Sal I (XM_001613146) and Belem (AF099662) strains deposited in GenBank. The primers used for the first round of PCR were 5′-TTCGCAACACTCGCCTTATTTCGCTCAACG-3′ and 5′-CCCCCAATTCGTCACCAATTTGTTTAGCAT-3′. The primers used for the nested PCR were 5′-TTTCGCTCAACGCGCGCATCTAAAATG-3′ and 5′-TTAGCATATTTTCTTCCGCCTCCTTTA-3′. The following thermal cycling conditions were used for both amplifications: 94°C for 5 min; 30 cycles of 94°C for 1 min, 52°C for 1 min and 72°C for 3 min, and a final extension at 72°C for 10 min. The PCR product was analysed on a 1.2% agarose gel, purified from the gel, and ligated into the T&A cloning vector (Real Biotech Corporation, Banqiao City, Taiwan). Each ligation mixture was transformed into *Escherichia coli* DH5α competent cells and positive clones with the appropriate insert were selected by colony PCR. The nucleotide sequences of the cloned insert were analysed by automatic DNA sequencing using the vector primers, M13 forward and M13 reverse primers. Sequencing analyses with two additional specific internal primers (5′-AGCAAAAACAGAAGCAGAAACAGCACA-3′ and 5′-GGAAATTTTCAGCTTCCGTTTTTGCTT-3′) were also performed to obtain the sequence of central region of the PvMSP-3β. At least two clones from each isolate were sequenced to ensure accuracy, and some isolates underwent three-fold sequence coverage to confirm the existence of rare polymorphisms. The nucleotide sequences reported in this study have been deposited in the GenBank database under the accession numbers JX667768-JX667772.

### Sequence and phylogenetic analysis of PvMSP-3β

The nucleotide and deduced amino acid sequences of PvMSP-3β were analysed using EditSeq and SeqMan in the DNASTAR package (DNASTAR, Madison, WI, USA). The phylogenetic tree was constructed using the neighbour-joining method in MEGA4 computational program [22]. Bootstrap proportions were used to assess the robustness of the tree with 1,000 bootstrap replicates. The coiled-coil motifs in each of the sequences were predicted using the Paircoil2 structural analysis program [23].

### Sequence polymorphism analysis

DNA sequence polymorphism analysis was performed on the 95 PvMSP-3β sequences. The number of segregating sites (S), haplotypes (H), haplotype diversity (Hd), nucleotide diversity (π), and the average number of pair-wise nucleotide differences within the population (*K*) were estimated using the DnaSP ver. 5.10.00 [24]. The π was calculated to estimate the step-wise diversity throughout the entire PvMSP-3β based on a sliding window of 100 bases with a step size of 25 bp. The rates of synonymous (dS) and non-synonymous (dN) substitutions were estimated and were compared using the Z-test (P <0.05) in MEGA4 program [22] using Nei and Gojobori’s method [25] with the Jukes and Cantor correction. To evaluate the neutral theory of evolution, the Tajima’s D test [26] was performed with the DnaSP ver. 5.10.00 [24]. Fu and Li’s D and F statistics [27] were also analysed using the DnaSP ver. 5.10.00 [24].

### Recombination parameters and linkage disequilibrium

The recombination parameter (R), which included the effective population size and probability of recombination between adjacent nucleotides per generation, and the minimum number of recombination events (Rm) were determined using the DnaSP ver. 5.10.00 [24]. The linkage disequilibrium (LD) between the different polymorphic sites was computed based on the R^2^ index.

## Results and discussion

**Figure 1:**
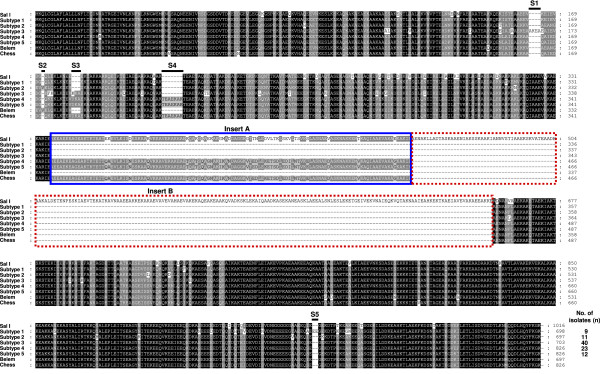
**Sequence polymorphism of the PvMSP-3β in Korean *Plasmodium vivax *isolates.** Sequence analysis of 95 *P. vivax* Korean isolates revealed five distinct subtypes of PvMSP-3β. The large-scale insert A and insert B are boxed with the blue line or dotted red line, respectively. The five small insertions (S1-S5) in the N-terminal and C-terminal domains are indicated with bold lines on the sequences. The total number of sequences for each subtype are listed in the right panel. The PvMSP-3β sequences of the Salvador I (Sal I), Belem and Chess were compared to those of Korean *P. vivax* isolates.

**Figure 2:**
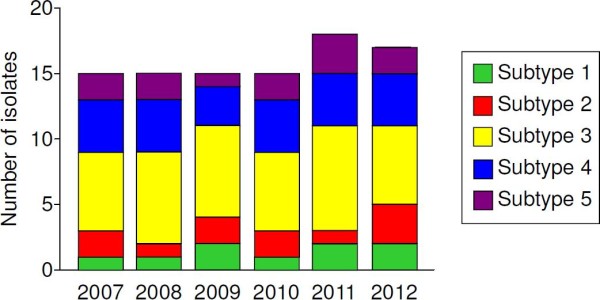
**Annual distribution of PvMSP-3β subtypes during the study period.** The 95 PvMSP-3β sequences from Korean isolates were analysed by year of collection.

**Figure 3:**
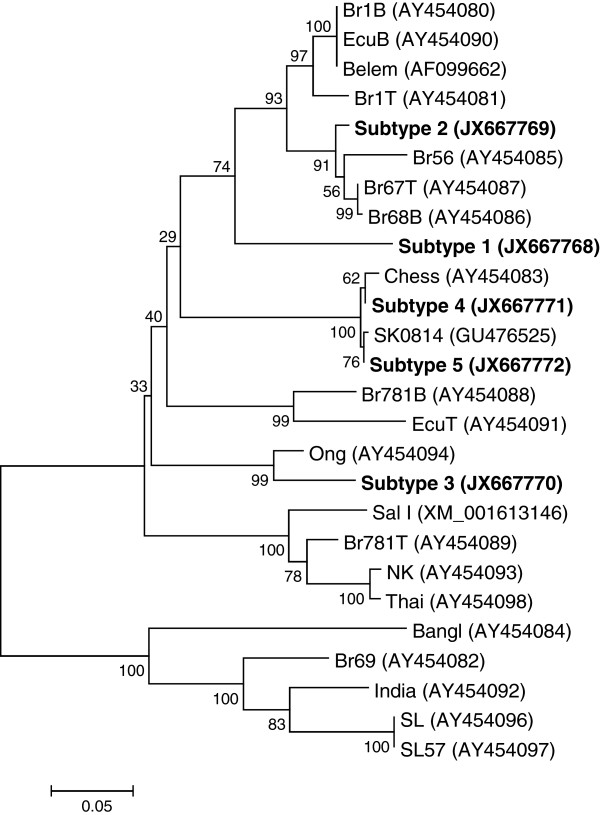
**Phylogenetic analysis of the PvMSP-3β in Korean *Plasmodium vivax *isolates.** The phylogenetic trees for the five PvMSP-3β Korean subtypes were constructed using a neighbour-joining method with the MEGA4 program. The numbers on the branches indicate the bootstrap proportions (1,000 replicates).

The full-length region encoding to PvMSP-3β was amplified from the genomic DNA from each of 95 *P. vivax* Korean isolates. The approximate sizes of amplified products were varied ranging from 2.1 to 2.5 kb. The nucleotide sequence analysis of the sequences revealed SNPs scattered throughout of the gene, as well as small or large insertions and deletions that resulted in variations in the size between the sequences. Based on the results of the sequence analysis, the 95 PvMSP-3β sequences of Korean isolates were classified as five different subtypes (subtypes 1, 2, 3, 4, or 5) (Figure 1). The N-terminal and C-terminal domains of PvMSP-3β are fairly polymorphic, containing SNPs and small insertions. Regarding the two large segments of insert A and insert B, which were found in Sal I sequence but not in Belem sequence [14], only two subtypes (subtypes 4 and 5) had a 120 amino acid-length insert A, but not a 200 amino acid-length insert B. The remaining three subtypes (subtypes 1, 2 and 3) had neither insert A nor B. Subtypes 2, 3, 4, and 5 contained small inserts in the N-terminal portion, whereas subtype 1 had a 2 amino acid-length small insert in C-terminal region. Subtype 3 was the most prevalent (n = 40), followed by subtype 4 (n = 23). No significant difference was observed in the annual prevalence of the subtypes (Figure 2). The phylogenetic analysis revealed that the Korean PvMSP-3β subtypes were clustered into five different clades, which differ to presently reported PvMSP-3β sequences (Figure 3). Interestingly, the Korean subtypes were widely distributed among different isolates from distinct geographic regions. This result differs from the previously reported result, which was that PvMSP-3β alleles tended to cluster based on geographic location of the isolates [14]. But, considering that sequence data for PvMSP-3β from worldwide isolates are scant, further complicated studies of *P. vivax* isolates from different geographical areas are required to characterize patterns in PvMSP-3β polymorphisms on a global scale.

**Figure 4:**
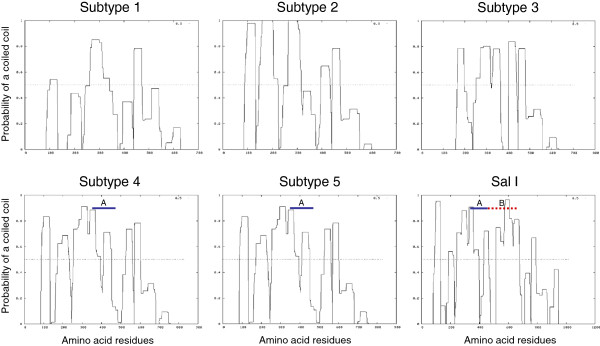
**Conservation of the coiled-coil tertiary structure.** The five subtypes of PvMSP-3β sequences of Korean *P. vivax* showed a high probability of forming coiled-coils. The portions corresponding to insert **A** and insert **B** are marked with the blue or dotted red lines, respectively.

Although the biological function of PvMSP-3β is not fully understood, the alanine-rich central core, an important structural feature found in its amino acid sequence, suggests that the protein forms a coiled-coil tertiary structure [4,5,14]. Based on its expression on the surface of merozoites, PvMSP-3β is predicted to be associated with other merozoite surface molecules, possibly through protein-protein interactions involving the coiled-coil structure [4-6], in a similar manner to PfMSP-3 [7]. These overall coiled-coil structures were well conserved in all Korean PvMSP-3β subtypes, even though central coiled-coil domains were more polymorphic than the N- and C-terminal regions, which are relatively conserved (Figure 4). These results are consistent with those of previous studies that the overall tertiary structure of PvMSP-3β is well conserved in *P. vivax* isolates despite sequence variation among the isolates [14].

**Figure 5:**
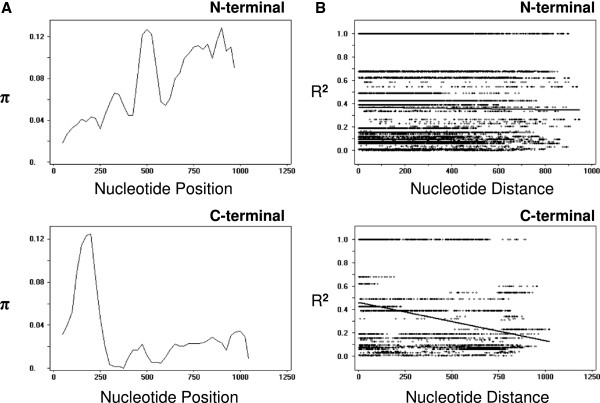
**Natural selection and recombination event in PvMSP-3β. (A)** The sliding window plot of nucleotide diversity per site (π) was constructed to compare the level of genetic diversity in the N-terminal and C-terminal domains of PvMSP-3β. The π values were calculated using the DnaSP with a window length of 100 bp and step size of 25 bp. **(B)** The linkage disequilibrium (LD) plot showed non-random associations between the nucleotide variants in 95 Korean *P. vivax* isolates at different polymorphic sites. The R^2^ values were plotted against nucleotide distance using a two-tailed Fisher’s exact test for statistical significance.

DNA sequence analyses were performed to examine the nucleotide diversity and genetic differentiation at PvMSP-3β among Korean *P. vivax* isolates. Nucleotide diversity was not consistently distributed within each domain or throughout the entire PvMSP-3β gene of Korean *P. vivax* isolates. The average number of pair-wise nucleotide differences (*K*) for the N-terminal domain of PvMSP-3β was 73.296 (Table 1). The overall haplotype diversity (Hd) and nucleotide diversity (π) for N-terminal domain was estimated to be 0.773 ± 0.028 and 0.07271 ± 0.00240, respectively (Table 1). The C-terminal domain of PvMSP-3β was more conserved than the N-terminal domain. The overall Hd and π for the C-terminal domain of PvMSP-3β was 0.733 ± 0.028 and 0.03040 ± 0.00179, respectively. Meanwhile, the insert A was highly conserved in subtypes 4 and 5, which represented a single haplotype. To examine whether natural selection had contributed to PvMSP-3β diversity within the Korean *P. vivax* population, the ratio of the average non-synonymous and synonymous mutation rates (dN/dS) in the PvMSP-3β sequences was analysed. The dN/dS ratio is widely used to evaluate the effects of natural selection on gene sequences. An excess of dN relative to dS (dN/dS ≥1) indicates positive selection, whereas a lack of dN relative to dS (dN/dS < 1) suggests negative or purifying selection imposed by functional constraints [25,28]. The estimated dN/dS for the N-terminal domain was 1.286, indicating that positive natural selection may be occurring in the N-terminal domain of PvMSP-3β of Korean *P. vivax* isolates (Table 1). The dN/dS value of the C-terminal domain was less than 1 (0.455), which suggesting purifying selection pressure may act on the domain. In order to more closely explore the effects of natural selection on each domain of PvMSP-3β, Tajima’s D test was applied. The Tajima’s D values for N-terminal domain and C-terminal domain of PvMSP-3β were 3.15439 (P <0.01) and 2.27420 (P <0.05), respectively, indicating both domains had been influenced by positive natural selection (Table 1). Analysis of the sliding window plot (window length 100 bp, step size 25 bp) showed that most of the nucleotide diversities found in the N-terminal domain occurred primarily in the middle and C-terminal ends of the N-terminal domain (Figure 5A). Meanwhile, in the case of C-terminal domain, the diversities were concentrated in the N-terminal end, decreasing toward the C-terminal end of the domain (Figure 5A). These findings are consistent with those of a previous report of unequally distributed nucleotide diversity within PvMSP-3β [14].

**Table 1:** DNA sequence polymorphisms in each domain of PvMSP-3β among Korean isolates

**Fragment**	**Segregating sites (S)**	**Singleton variable sites**	**Parsimony informative sites**	**Total no. of mutations**	***K***	**H**	**Hd ± SD**	**π ± SD**	**dN-dS**	**Tajima’s D**	**Fu and Li’s D**	**Fu and Li’s F**
N-terminal domain	187	0	187	194	73.296	5	0.773 ± 0.028	0.07271 ± 0.00240	1.286	3.15439 (P < 0.01)	2.53522 (P < 0.02)	3.36192 (P < 0.02)
Insert A	0	0	0	0	0	1	0	0	0	0	0	0
C-terminal domain	96	0	96	100	32.920	5	0.733 ± 0.028	0.03040 ± 0.00179	0.455	2.27420 (P < 0.05)	2.39279 (P < 0.02)	2.81226 (P < 0.02)

*K*, average number of pairwise nucleotide differences; H, number of haplotypes; Hd, haplotype diversity; π, observed average pairwise nucleotide diversity; dN, rate of non-synonymous mutations; dS, rate of synonymous mutations.

Based on the occurrence of small indels in the sequence and the presence of degenerate repeat on either side of the indels, DNA polymerase slippage has been suggested as a possible mechanism generating diversity of PvMSP-3β [14]. Similar small indels and flanking degenerate repeats were also identified in PvMSP-3β sequences of Korean *P. vivax* isolates, which suggested slipped-strand mispairing during DNA replication may have contributed to PvMSP-3β diversity. The 11 amino acid-length repeated elements that were identified in the large-scale insertion [14] were also conserved in the end of the N-terminal domain and the end of insert A of PvMSP-3β of Korean *P. vivax* isolates. The diversity of *Plasmodium* antigens, including PvMSP-3α, is also known to be generated by intragenic recombination events, and is maintained by balancing selection [9,10,20,28]. Similarly, it has been postulated that recombination is another major factor generating allelic diversity of PvMSP-3β [14]. To understand the role of the recombination event in PvMSP-3β in Korean *P. vivax* isolates, the recombination parameters and the linkage disequilibrium in the gene were analysed. For the N-terminal domain of PvMSP-3β, the minimum number of recombination events between adjacent polymorphic sites (Rm) was 13, whereas the R between adjacent sites (Ra) and per gene (Rb) was 0.0036 and 3.6, respectively. In the case of C-terminal domain of PvMSP-3β, the Rm was 4 with a Ra of 0.0029 and a Rb of 3.1. These high recombination parameter values suggested that recombination may have occurred between sites, contributing to genetic diversity in PvMSP-3β gene. The linkage disequilibrium index, R^2^, was also declined across the analysed region, which further indicates that intragenic recombination might have contributed to the diversity observed in both the N-terminal and C-terminal domains of PvMSP-3β in Korean *P. vivax* isolates (Figure 5B).

The genetic diversity of malaria parasites is closely associated with the levels of endemicity and intensity of transmission [29,30]. The *P. vivax* population in hyperendemic areas, such as Papua New Guinea, is highly diverse and shows multiplicity of infections [31,32]. However, complex genetic structures have also been identified in *P. vivax* populations in hypo-endemic areas, such as China, Myanmar and Thailand [10,33-38]. Several recent studies have suggested that genetic diversity within the *P. vivax* population in Korea has increased in recent years [17-21]. Considering the low endemicity and transmission intensity in Korea, it remains unclear how genetic diversity of *P. vivax* in Korea is rapidly disseminated and maintained in recent years. An increased influx of international travellers and foreign workers might have contributed to the increasing allelic variation in the Korean *P. vivax* population through the introduction of malaria parasites from other endemic areas [17]. It is also possible that the Korean *P. vivax* population has evolved under evolutionary pressure, most probably related to host immune response. Further studies of genetic diversity in the Korean *P. vivax* population in Korea are warranted to clarify the patterns of genetic variation and the biological relevance of increasing *P. vivax* diversity.

## Conclusions

Radical genetic polymorphism was identified in the PvMSP-3β gene of Korean *P. vivax* isolates. The size of the PvMSP-3β varied, as a result of large and small insertions/deletions, and numerous SNPs were observed throughout the gene. Despite the high level of sequence diversity, the coiled-coil tertiary structure of the protein was well conserved in all of the Korean *P. vivax* isolates, suggesting that the protein is under functional constraints. The genetic diversity of the PvMSP-3β of Korean *P. vivax* isolates has been influenced by natural selection, particularly in the N-terminal and C-terminal domains. Polymerase slippage during DNA replication and intragenic recombination might have contributed to the genetic diversity of PvMSP-3β in the Korean *P. vivax* population. These findings of parasite heterogeneity are relevant to the development of a PvMSP-3β-based vaccine against *P. vivax* and the implementation of malarial control programmes in Korea.

## Competing interests

The authors declare that they have no competing interests.

## Authors’ contributions

JMK and HLJ performed all the experiments and analysed the sequence data. PYC, SKA and SUM collected blood samples or performed sequence and phylogenetic analyses. BKN and TSK designed the study and supervised the study process. JMK and BKN wrote the paper. TSK, HWL and WMS assisted in writing and editing the manuscript. All authors read and approved the final manuscript.
